# Genomic analysis of *Streptococcus canis* from different hosts in Italy 2004–2021: diversity, antimicrobial resistance, and virulence profiles

**DOI:** 10.3389/fvets.2025.1645885

**Published:** 2025-08-19

**Authors:** Lucilla Cucco, Elisa Albini, Francesca Blasi, Serenella Orsini, Alessandro Fiorucci, Annalisa Dettori, Sara Petrin, Arianna Peruzzo, Silvano Salaris, Marta Paniccià, Giovanni Pezzotti, Francesca Romana Massacci, Chiara Francesca Magistrali

**Affiliations:** ^1^Istituto Zooprofilattico Sperimentale dell'Umbria e delle Marche "Togo Rosati", Perugia, Italy; ^2^Istituto Zooprofilattico Sperimentale delle Venezie, Padova, Italy; ^3^Istituto Zooprofilattico Sperimentale della Lombardia e dell'Emilia Romagna "Bruno Ubertini", Brescia, Italy

**Keywords:** *Streptococcus canis*, antibiotic resistance, virulence, zoonosis, epidemiology

## Abstract

*Streptococcus canis*, a multi-host pathogen commonly isolated from dogs and cats has been occasionally reported in severe cases of human infection. This study aimed to explore the genetic diversity, antimicrobial resistance (AMR), and pathogenicity of *S. canis* isolates collected between 2004–2021, in Italy. Fifty-five *S. canis* isolates from clinical cases in domestic animals were investigated for susceptibility to antibiotics and then characterized for sequence type (ST), virulence profile, and antimicrobial-resistant genes through whole genome sequencing (WGS). All isolates were susceptible to beta-lactams, while frequently exhibiting resistance to lincosamides, chlortetracyclines, and macrolides. Six out of 55 isolates of *S. canis,* all collected between 2020 and 2021, were multi-drug resistant (MDR). The most common AMR gene in the dataset was *lmrP* conferring resistance for streptogramin, tetracycline, macrolide, streptogramin A, and lincosamide. Other determinants of AMR were the *tet* genes. Twenty-one distinct STs were identified, with ST9 being the most prevalent in our collection. Regarding the virulence genes, forty-three isolates were positive for the *ssp-5* gene, which encodes an agglutinin receptor. Comparison with other 46 *S. canis* genomes available in public repositories revealed that the Italian isolates clustered by the *S. canis* M-like (SCM) protein gene and ST and did not group according to their host, area, or year of origin. In conclusion, our study underscores the susceptibility of Italian *S. canis* isolates to beta-lactam antibiotics, which remain the first line of defense in managing infections. In Italy, ST9 represents the predominant clone of this pathogen. Despite the diversity in species of origin and the various STs identified, our findings confirm that *S. canis* has not adapted to different ecological niches and corroborate the accidental pathogenic nature of human cases.

## 1 Introduction

*Streptococcus canis* is a *β*-hemolytic *Streptococcus* species from the Lancefield Group G, typically colonizing the skin and mucous membranes of asymptomatic dogs and cats (1, 2). *S. canis* can cause a variety of infections in these animals, including skin and soft tissue infections, and, although rare, more severe diseases such as ulcerative keratitis, necrotizing fasciitis, septicemia, endocarditis, respiratory disease, genital, and urinary infections (3–8). Less frequently, *S. canis* has been found in other wild and domestic mammalian hosts: in cattle is a rare but contagious agent of mastitis (3, 9). *S. canis* is also a zoonotic agent, with the first infection in humans described in 1998. Since then, a growing number of cases have been reported in humans, including severe cases of bacteremia, osteomyelitis, endocarditis, and pneumonia (10–12). Human infections are generally a consequence of exposure to dogs, or, less frequently, to cats, and occur after bite wounds, superficial ulcers, or cellulitis (12). Molecular epidemiological investigations have revealed significant genomic overlap between animal and human isolates, suggesting direct interspecies transmission (5, 13). Moreover, *S. canis* infection is rare in the setting of neonatal sepsis; however, it can lead to high morbidity and mortality as reported recently (14).

Therapy against *S. canis* infection is based on antimicrobials, with penicillins identified as the first-line antibiotic class in animals and humans (15). The development of AMR in *S. canis* has been reported, but the mechanisms of resistance are not yet fully characterized (16–18). *S. canis* has been reported to show low resistance rates to quinolones (7.0%) and from 5.6 to 39.7% for tetracyclines (15). Conversely, the species is considered highly susceptible to beta-lactams (15). This assumption has recently been challenged by reports of beta-lactam-resistant isolates from animals emerging in Japan (19).

Despite some research efforts, *S. canis* remains less studied compared to other streptococcal species, and many aspects of its epidemiology and virulence are still not well understood (3–5, 20). Two genotyping methods, a multi-locus sequence typing (MLST) scheme (21) and a scheme based on the allelic variations of the SCM protein gene (17), were used to investigate the diversity of *S. canis* population. Nevertheless, a consensus on the preferred method for *S. canis* typing was not reached, complicating the understanding of the population structure of this pathogen (13, 21–23). More recently, these two systems were compared to core genome typing based on data from WGS (5). This comparison highlighted that both MLST and SCM typing schemes lack in describing the diversity within the *S. canis* population, probably because they both analyze small fragments of the bacterial genome. By contrast, core genome analysis based on WGS provides a more comprehensive understanding of the epidemiology of this pathogen (5, 13). The application of WGS could potentially address many of the current knowledge gaps regarding *S. canis*, particularly in terms of its population structure, evolution, and host specificity.

Here we use whole genome sequencing of *S. canis* isolates collected between 2004 and 2021 from clinical cases in domestic animals in Italy to investigate genetic diversity, antimicrobial resistance, and pathogenicity.

## 2 Materials and methods

### 2.1 Bacterial isolates and species identification

We investigated 55 isolates collected from dogs (*n* = 25), cats (*n* = 13), cattle (*n* = 3), and a hedgehog (*n* = 1) collected in Italy from 2004 to 2021 (Supplementary Table S1). All the isolates originated from individual cases of clinical disease. To the best of our knowledge, they were not epidemiologically linked, as they were collected from different animals, in different geographical locations, at different years, and from different veterinary clinics or diagnostic laboratories. In our collection, we included the reference strain of Culture Collection University of Gothenburg (CCUG): *Streptococcus canis* CCUG 27668. The samples were cultured on 5% sheep blood agar (Biolife Italiana Srl, Milan, Italy) at 5% CO_2_, 37°C for 24–48 h. Suspected *β*-hemolytic colonies were selected, and confirmed as belonging to the genus *Streptococcus* by Matrix-Assisted Laser Desorption Ionization-Time of Flight Mass Spectrometry (MALDI-TOF MS) (Bruker Daltonics GmbH, Germany).

Isolates were identificated at specie level by the 16S rRNA gene sequencing. DNA was extracted using QIAamp® DNA Mini Kit (Qiagen, Hilden, Germany) following the manufacturer’s instruction and used to perform the PCR reaction with universal primers 27F (5′-AGAGTTTGATCCTGGCTCAG-3′) and 1492R (5′-TACGGYTACCTTGTTACGACTT-3′) (24), containing 10 μL of 5x Taq buffer, 1.5 mM MgCl_2_, 200 μM dNTPs, 1 U of Taq DNA polymerase (Promega Corporation, Wisconsin, USA), 0.2 μM of primers and 10 ng of DNA template, brought up to a final volume of 50 μL with ultra-pure water. The reactions were performed on a thermocycler (Eppendorf, Hamburg, Germany) under these conditions: 4 min at 96°C, followed by 30 cycles of 1 min at 94°C, 1 min at 56°C and 1 min at 72°C, and a final extension step at 72°C for 10 min. Aliquots of 5 μL of each reaction were analyzed on 1% (w/v) agarose gel in TBE buffer.

The PCR products were purified using High Pure PCR Product Purification Kit (Roche, Basel, Switzerland), sequenced with specific primers using BigDye™ Terminator v3.1 Cycle Sequencing Kit (Applied Biosystems, Massachusetts, USA) in 3500 Genetic Analyzer (Applied Biosystems Massachusetts, USA). The DNA sequences were analyzed using BioEdit sequence alignment tool and compared with the sequences deposited in the National Center for Biotechnology Information (NCBI)-GenBank database using the BLAST alignment tool.^1^ The isolates were identified unambiguously, with ≥ 98.7% similarity to the 16S rRNA sequence of the corresponding strain.

### 2.2 Antimicrobial susceptibility testing

We assessed MICs using a commercial MIC panel (BOP06F, Sensititre; Trek Diagnostic Systems Inc.) according to the manufacturer’s instructions. Susceptibility to erythromycin was assessed using Etest strips (Liofilchem, Roseto degli Abruzzi, Italy), with a tested concentration range of 0.016–256 μg/mL. *Streptococcus pneumoniae* ATCC 49619 was used as a quality control strain. The MIC values for chlortetracycline, penicillin, trimethoprim/sulfamethoxazole, and erythromycin were interpreted using the breakpoints recommended by the Clinical Laboratory Standards Institute M100 Ed. 34th (25). MIC values for ampicillin, clindamycin, ceftiofur, and enrofloxacin were interpreted according to the breakpoints from CLSI Vet01S, Ed. 7th edition (26). Based on the clinical breakpoints, the isolates were classified as susceptible (S), intermediate (I), or resistant (R). An isolate was classified as multi-resistant when it was resistant to at least three antibiotic classes representing third-generation cephalosporins (ceftiofur), penicillin (ampicillin and penicillin), lincosamides (clindamycin), fluoroquinolones (enrofloxacin), sulfonamides (trimethoprim/sulfamethoxazole), macrolides (erythromycin), and tetracycline (chlortetracycline) (27). For gentamicin and florfenicol, MIC values were interpreted using the epidemiological cut-off (ECOFF) criteria established by EUCAST.^2^ When using the ECOFF, the isolates were categorized as wild-type (WT) or non-wild-type (nWT). According to EUCAST definitions, “wild-type” (WT) isolates are those with MICs at or below the epidemiological cutoff value (ECOFF), representing populations without acquired or mutational resistance mechanisms, whereas “non-wild-type” (NWT) isolates exhibit MICs above the ECOFF, indicating the likely presence of such resistance mechanisms.

### 2.3 Whole genome sequencing

In order to investigate ST, virulence profile, and antimicrobial resistant genes, the 55 *S. canis* isolates were whole genome sequenced. Each DNA was then quantified with the Qubit fluorometer (QubitTM DNA HS Assay, Thermo Fisher Scientific Inc.). Libraries were prepared using the Nextera XT Library Prep kit (Illumina Inc., San Diego, CA) and then sequenced on an Illumina NextSeq 550 platform to generate 300 bp paired-end reads.

### 2.4 Bioinformatic analysis

Illumina reads were trimmed and checked for quality using Fastp v0.19.5 (28) with default parameters. The metrics used for reads quality assessment were: number of contigs, mean values for N50 and L50 and GC% values (Supplementary Table S2). The reads were assembled using SPAdes genome assembler v3.11.1 (29), checked for quality assessment of draft genome sequences with QUAST v5.0.2 (30), and annotated using Prokka v1.14.6 (31). The resulting general feature formats (GFFs) produced by Prokka were analyzed with Roary v3.11.3 (32) to obtain a core genome alignment. *In silico* multi-locus (ML) ST analysis was performed by submitting sequences to *S. canis* MLST database to obtain allele number and ST. The new allele sequences or STs were submitted to the database curator.^3^

Antibiotic-resistance genes were analyzed with ABRicate,^4^ using ResFinder, considering only genes with a ≥95% coverage and ≥99% identity.

Our investigation of quinolone resistance focused on *parE*, *gyrB*, *parC,* and *gyrA* mutations. *parE*, *gyrB*, *parC*, and *gyrA* sequences of the 55 isolates were manually aligned running MUSCLE online^5^ and using *Streptococcus canis* HL_98_2 (GenBank NZ_CP053789.1) as reference.

Virulence genes were searched by BLASTN v2.13.0+ creating a database of 19 previously described genes (5) and using a ≥90% coverage and ≥20% identity.

In order to compare our isolates to the ones available in the literature, we downloaded the 46 genomes referring to the *S. canis* (5), in the Sequence Read Archive (SRA) database. The dataset included 26 isolates from dogs, 11 from humans, 6 from cats, 2 from cattle, and 1 from seal. Those selected isolates were originated from UK (41), South Korea (4) and USA (1). The 46 genomes were compared with the 55 isolates of our study creating a maximum likelihood (ML) phylogenetic tree (FastTree 2.1.11) (33) of the core genome of *S. canis* isolates. The tree was manually annotated using iTOL (v.6, https://itol.embl.de/, accessed date: September 2, 2024).

We classified SCM 1-15 using BLASTN and a database of SCM coding sequences previously described by Pagnossin et al. (5). For the classification, we chose an identity percentage >98% and a coverage percentage >70% (5). The nucleotide sequences of the M protein gene of our *S. canis* isolates were aligned with the nucleotide sequences of M proteins available in the literature using MUSCLE (5).

## 3 Results

### 3.1 AMR phenotypes and genotypes

**Figure 1:**
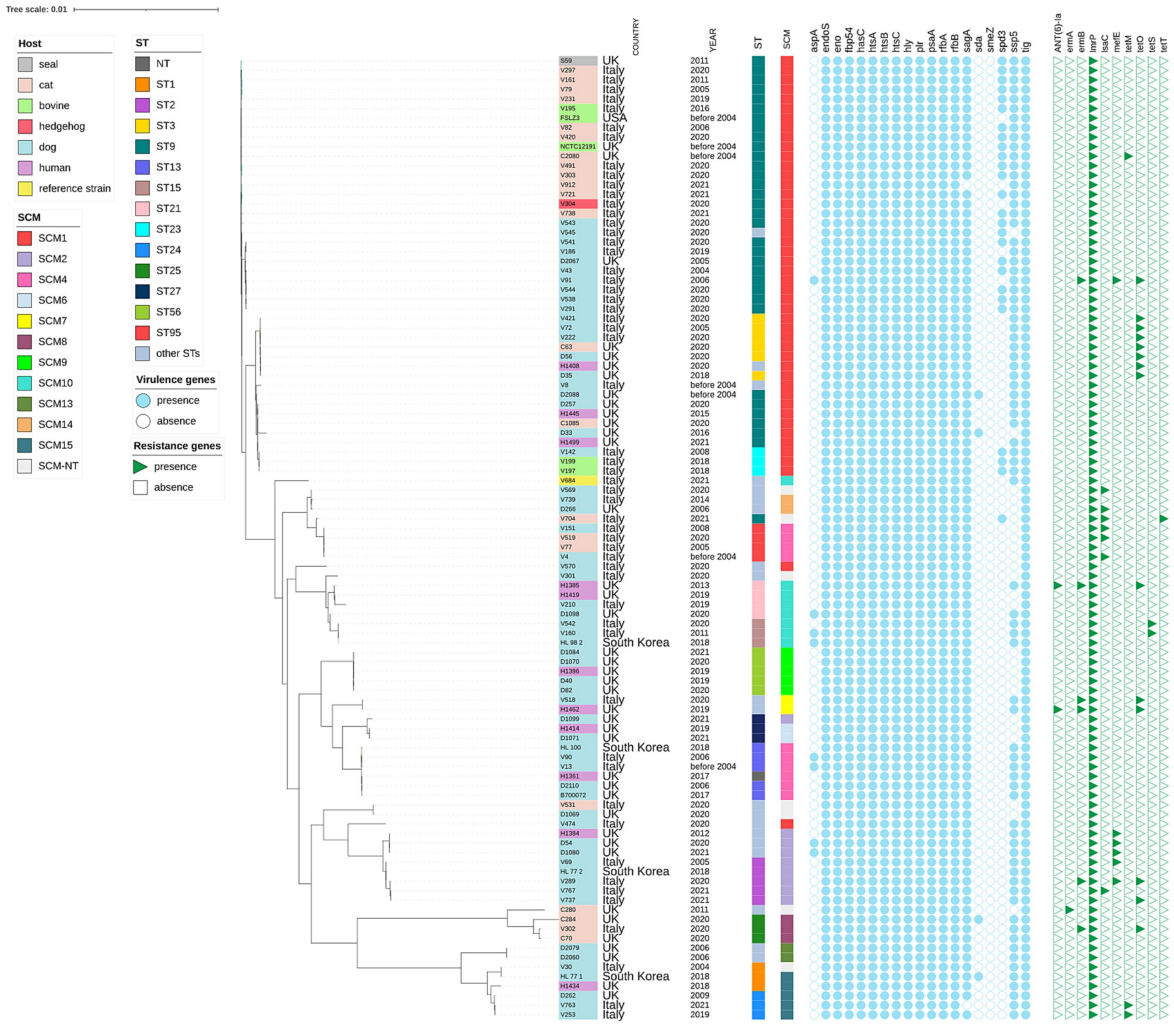
Maximum likelihood phylogenetic tree based on core genome alignment of 101 *Streptococcus canis* isolates collected from various countries and hosts. The tree was constructed and annotated using the iTOL interactive interface (https://itol.embl.de). For each isolate, the sequence type (ST), SCM group, virulence genes, and antibiotic resistance genes are indicated. The isolate labels are color-coded according to the host of origin (e.g., dog, cat, seal, human, bovine, hedgehog), enabling immediate visual correlation between host specificity and phylogenetic clustering.

The distribution of *S. canis* isolates according to antibiotic MIC values is shown in Table 1. The isolates were classified as resistant to chlortetracycline (18/55, 32.7%), clindamycin (6/55, 10.9%) and to erythromycin (6/55, 10.9%). Forty-five isolates out of 55 (81.8%) were intermediate for enrofloxacin. Moreover, 41/55 (74.5%) isolates were considered as non wild-type for gentamicin. Multi-resistance was detected in 6/55 (10.9%) isolates of *S. canis,* showing simultaneous resistance to erythromycin, chlortetracycline and clindamycin. Those isolates, collected between 2020 and 2021, belonged to dogs (4), and cats (2). The most common AMR gene in the dataset was *lmrP* (55/55) conferring resistance for streptogramin, tetracycline, macrolide, streptogramin A, lincosamide. Other determinants of AMR were the *tet* genes (Table 2, Figure 1).

**Table 1:** Distribution of MIC (minimum inhibitory concentration) values among the 55 *S. canis* isolates tested using a commercial MIC panel (BOP06F, Sensititre; Trek Diagnostic Systems Inc.).

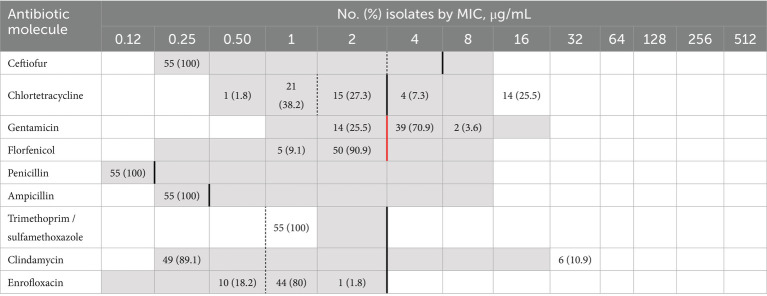

Percentages are shown in brackets. The grey-shaded areas indicate the range of concentrations actually tested for each antibiotic, for which interpretive criteria were available. Black vertical bars indicate the threshold values for clinical resistance, black dotted lines indicate the threshold values for intermediate susceptibility according to Clinical and Laboratory Standards Institute (https://clsi.org). Red vertical bars indicate the threshold values for the epidemiological cut-off values, according to The European Committee on Antimicrobial Susceptibility Testing (https://www.eucast.org/).

**Table 2:** Antimicrobial resistance genes and their associated classes of antibiotics, as indicated by the Comprehensive Antibiotic Resistance Database (CARD; https://card.mcmaster.ca/home), identified in 55 *Streptococcus canis* isolates.

Antimicrobial resistance genes	Number of isolates (%)	Associated classes of antibiotics
*lmrP*	55 (100)	streptogramin, tetracycline, macrolide, streptogramin A, lincosamide
*tet(O)*	8 (14.5)	tetracycline
*lsaC*	6 (10.9)	pleuromutilin, streptogramin, lincosamide
*ermB*	4 (7.3)	streptogramin, macrolides, streptogramin A, streptogramin B, lincosamide
*mefE*	3 (5.4)	macrolide
*tet(S)*	2 (3.6)	tetracycline
*tet(M)*	2 (3.6)	tetracycline
*tet(T)*	1 (1.8)	tetracycline

None of the isolates harbored the mutations associated to a reduced susceptibility to quinolones, namely Ser81/Glu85 in *gyrA*, Gly408 in *gyrB*, Ser67/Asp71 in *parC* or Asp438 in *parE* (34) (Supplementary Table S3).

### 3.2 Genomic analysis

The mean length of the 55 assemblies was 2,099,785 (min 1,892,070; max 2,486,022) with an average number of contigs of 76 (min 45; max 162). The mean values for N50 and L50 were 84,699 (min 29,080; max 155,133) and 9 (min 5; max 23). GC% value ranged between 39.52 and 38.82 (Supplementary Table S2). Twenty-one distinct STs were identified, with ST9 being the most prevalent, accounting for 38.2% (*n* = 21) of our collection (Supplementary Table S4). Both ST95 and ST2 were also common, each representing 7.3% (*n* = 4) of the samples. Eight new STs were identified as ST91-ST101 (ID289-ID299).^6^

The distribution of putative virulence genes was investigated in the 55 *S. canis* isolates and we described the presence of 18 out of 19 virulence genes as reported in Supplementary Table S1 and Figure 1. Twelve of these genes (63.2%) were detected in each isolate. Forty-three isolates (78.8%) of our collection were positive for the carriage of the *ssp-5* gene, which encodes an agglutinin receptor. None of our isolates were positive for *smeZ*.

The characterization of the allelic variations of the SCM gene across the collection is provided in Supplementary Table S1 and Figure 1.

### 3.3 Phylogenetic analysis

Comparison with other *S. canis* genomes available in public repositories revealed that the Italian isolates clustered by the SCM and ST. Some STs belonged to one SCM allele alone: e.g. ST1 to SCM15, ST3 to SCM1, ST15 to SCM10, ST23 to SCM1 (Figure 1, Supplementary Table S1). The agreement among phylogenetic methods was not complete: in the ML phylogenetic tree, ST3, ST23, ST61, ST94, and ST101 clustered together with ST9, while all of them were classified as SCM1. This ST9-SCM1 phylogenetic cluster was the largest in our collection. Regardless of the typing method, the phylogenetic tree indicated that the 101 *S. canis* isolates did not group according to their host, area or year of origin. Human isolates belonged to different STs, harbored various SCM types and were scattered along the phylogenetic tree (Figure 1).

## 4 Discussion

*S. canis* is a significant pathogen in both canines and cattle and is increasingly recognized as an emerging zoonotic agent. Despite its clinical importance, there remains a scarcity of comprehensive data regarding the epidemiology, antibiotic susceptibility, and virulence mechanisms of this bacterium. Our study addresses this gap by presenting data on *S. canis* isolates collected from various animal hosts over a span of 17 years in Italy. Additionally, we conducted comparative genomic analyses between our *S. canis* isolates and publicly available genomes from human cases and different geographical regions, thereby providing new insights into the genetic diversity of this microorganism.

Regarding the analysis of virulence characteristics, we identified 18 out of the 19 virulence genes previously described in the literature (5). Several genes such as *eno, fbp54, hasC, hyl, plr, rfbA, rfbB*, which were identified in our collection, are recognized as components of the *S. pyogenes* core genome, which further supports the close evolutionary relationship between *S. canis* and *S. pyogenes* (35). *hasC* is part of the *has* operon, which is responsible for the synthesis of hyaluronic acid. In fact, in *S. pyogenes*, the capsule composed of hyaluronic acid has a composition analogous to that of hyaluronic acid found in human connective tissue, which contributes to the low immunogenicity of the bacterium in the host (36). In the genus *Streptococcus*, *fbp54* encodes a surface protein capable of binding to fibrinogen and fibronectin, thus being involved in adhesion mechanisms (37). Forty-three isolates were positive for the ssp-5 gene, being one of the most prevalent virulence genes in our collection. The gene *ssp-5* codes for an agglutinin receptor and is responsible for adhesion and colonization of *Streptococcus* to different substrates inside the host (38). Notably, ssp-5 has been identified in other *Streptococcus* species, such as *S. suis* and *S. canis*, both of which are associated with zoonotic transmission from animals to humans. Its presence is strongly correlated with increased pathogenicity, making it a critical factor in cross-species infections and a valuable target for surveillance and therapeutic interventions (5, 38).

Moreover, *S. canis*, like the majority of species within this genus, is generally susceptible to the beta-lactam class of antibiotics (4, 39, 40). A recent study has reported reduced susceptibility to penicillin-G in *S. canis* isolates from dogs in Japan, with this resistance attributed to amino acid substitutions in penicillin-binding proteins (15). In contrast, all isolates in our collection exhibited full susceptibility to beta-lactams, including penicillin, ampicillin, and ceftiofur. This finding reinforces the continued efficacy of beta-lactams as the first-line treatment for *S. canis* infections in Italy. Our isolates frequently exhibited resistance to lincosamides, tetracyclines, and macrolides consistent with previous reports (4, 5, 41–43). Resistance to tetracyclines was detected in approximately one-third of our collection, a proportion aligning with other studies, where it ranges from 30–40% (4, 21, 44, 45). This resistance was associated with the presence of *tet* genes, with *tet*(O) being the most prevalent, followed by *tet*(M), *tet*(S), and *tet*(T). While the presence of *tet*(O) and *tet*(M) is well documented in the literature, the detection of *tet*(S) and *tet*(T) is relatively rare (5, 46). Notably, all Italian ST15 isolates harbored *tet*(S), and all ST3 was positive for *tet*(O).

Resistance to macrolides and lincosamides, likely attributable to the MLSB phenotype (macrolides, lincosamides, and streptogramin B group), was detected in six isolates. This resistance was generally associated with the presence of the *lmrP* and *ermB* determinants (4, 42). Additionally, up to 80% of the isolates were categorized as non-susceptible to enrofloxacin according to the CLSI breakpoints for veterinary pathogens, which classify MIC values of 1–2 μg/mL as intermediate. In *S. canis*, resistance to quinolones is generally associated with substitutions in the *parC* (Ser67/Asp71), *gyrA* (Ser81/Glu85) and *parE* (Asp438) sequences (47). None of these substitutions were detected in our collection (Supplementary Table S3). It is noteworthy that the aforementioned substitutions are typically coupled with MIC values for quinolones higher than 2 μg/mL, while our isolates exhibited MIC values equal or below 2 μg/mL (47).

A total of 6 out of 55 *S. canis* isolates (10.9%) obtained from the bacterial collection between 2020 and 2021 exhibited a multi-drug resistant phenotype. Of these, four isolates originated from canine hosts, three of which belonged to ST2, while the remaining two were derived from cats. The emergence of MDR strains within *S. canis* is a growing concern, as resistance to multiple antibiotic classes can severely limit therapeutic options for treating infections in companion animals. The observed association of MDR with ST2 strains may indicate clonal expansion or selective pressure within this lineage. From a clinical standpoint, MDR *S. canis* infections may result in prolonged illness, treatment failure, or increased reliance on last-resort antimicrobials. Moreover, the potential zoonotic transmission of resistant *S. canis* strains from pets to humans—particularly immunocompromised individuals—poses a notable public health risk, as companion animals can act as reservoirs and vectors for antimicrobial-resistant bacteria (13). These findings underscore the importance of routine antimicrobial susceptibility monitoring and the implementation of prudent antibiotic use policies in veterinary practice.

There is no standard reference technique for the phylogenetic analysis of *S. canis*; therefore, three methods were utilized in parallel: core genome analysis, MLST and SCM sequences analysis. For the phylogenetic analysis, publicly available genomes of *S. canis* were also included, even though their number was quite limited. The data confirm that ST9 of *S. canis* is a dominant sequence type in Italy, consistent with previous studies in other European countries, such as Portugal and Germany (48). ST9 is characterized by the presence of allele 1 of the *S. canis* M-like protein (SCM1), a recognized virulence factor of this bacterium. Similarly, ST21 was associated with the production of SCM allele 10, as previously reported by Fukushima et al. (49), but this association is not exclusive, as the same variant was found in ST15. The phylogenetic analysis of the core genome revealed that the isolates did not cluster based on their species of origin. For instance, isolates belonging to ST9 originated from diverse sources, including dogs, cats, cattle, hedgehogs, and seals, yet were placed within the same clusters in the phylogenetic tree. Human-origin isolates did not form separate clusters but were included within the same clusters as canine, feline, and bovine isolates. The comparison among the three methods highlights an incomplete agreement between MLST typing and the core genome analysis, as shown by the presence of multiple STs in the same cluster. Genomic analyses, including multilocus sequence typing, confirm the zoonotic origin of these infections and illustrate genetic recombination events with *Streptococcus dysgalactiae*, enhancing its virulence and adaptability (21). ST9 was isolated from a patient with bacteremia, while previous animal studies had consistently identified ST9 in dogs suffering from dermatitis and wound infections (22). This suggests that certain STs are predisposed to cross-species infection and may possess enhanced virulence factors, such as the *scm* gene.

The SCM classification system groups *scm* alleles based on sequence similarity into three major categories: Group I (alleles 1–7), Group II (alleles 8–15), and SCM-NT (non-typeable). Group I includes classical alleles typically associated with less invasive strains, while Group II encompasses novel variants such as allele 10, which has been linked to increased intracellular invasion and potential virulence, particularly in strains belonging to ST21 and ST15 (49). SCM-NT strains either lack detectable *scm* sequences or express untypeable variants, and their role in pathogenesis is still under investigation. This grouping provides a molecular framework for epidemiological and virulence profiling of *S. canis*, particularly in zoonotic contexts (49, 50). We did not observe a clustering between SCM group 1 isolates and SCM group 2 isolates in the phylogenetic tree. As already noted by Pagnossin et al. (5), the analysis of data from WGS offers higher discrimination as compared to SCM or MLST analysis. Our study provides *S. canis* genomes from novel geographical regions, periods, and hosts, thereby offering new opportunities to compare this pathogen diversity across various ecological niches.

The high genetic similarity of *S. canis* isolates from different hosts and tissues confirms the generalist nature of this pathogen and its lack of adaptation to specific host species, in agreement with findings by other authors (5, 13). A limitation of this study is the relatively small number of *S. canis* isolates analyzed. However, the strains were collected over a broad temporal span (2004–2021) and from diverse host species and geographic regions, which enhances the relevance of the observed phylogenetic patterns and allows for insights into potential long-term and cross-host transmission dynamics. Regardless of genetic lineage, *S. canis* seems capable of cross-species transmission. This genomic evidence is supported by the nature of infections reported in cattle, where before spreading among lactating cows, *S. canis* infection usually originates from cats or dogs having access to the barn (9). More importantly, *S. canis* infections in humans are primarily attributed to a close contact, often through bites, with dogs and cats (4).

In conclusion, our study underscores the susceptibility of Italian *S. canis* isolates to beta-lactams antibiotics, which remain the first line of defense in managing infections. In Italy, ST9 represents the predominant clone of this pathogen. Despite the diversity in species of origin and the various sequence types identified, our findings confirm that *S. canis* has not adapted to different ecological niches, corroborating the accidental pathogenic nature of human cases.

## Data Availability

The data presented in the study are deposited in the National Center for Biotechnology Information SRA [BioProject ID PRJNA1175870 (accession numbers SAMN44373913—SAMN44373967)].

## Footnotes

^1^ www.ncbi.nlm.nih.gov/BLAST

^2^ https://www.eucast.org/

^3^ https://pubmlst.org/organisms/Streptococcus-canis

^4^ https://github.com/tseemann/abricate

^5^ https://www.ebi.ac.uk/Tools/msa/muscle/

^6^ https://pubmlst.org/organisms/Streptococcus-canis

## References

[1] Devriese LA Hommez J Kilpper-Balz R Schleifer KH. *Streptococcus canis* sp. nov.: a species of group G streptococci from animals. Int J Syst Bacteriol. (1986) 36:422–5. doi: 10.1099/00207713-36-3-422

[2] Fukushima Y Tsuyuki Y Goto M Yoshida H Takahashi T. Species identification of β-hemolytic streptococci from diseased companion animals and their antimicrobial resistance data in Japan (2017). Jpn J Infect Dis. (2019) 72:94–8. doi: 10.7883/yoken.JJID.2018.231, PMID: 30381681

[3] Hassan AA Akineden Ö Usleber E. Identification of *Streptococcus canis* isolated from milk of dairy cows with subclinical mastitis. J Clin Microbiol. (2005) 43:1234–8. doi: 10.1128/JCM.43.3.1234-1238.2005, PMID: 15750089 PMC1081216

[4] Pagnossin D Smith A Oravcová K Weir W. *Streptococcus canis*, the underdog of the genus. Vet Microbiol. (2022) 273:109524. doi: 10.1016/j.vetmic.2022.109524, PMID: 35933975

[5] Pagnossin D Weir W Smith A Fuentes M Coelho J Oravcova K. *Streptococcus canis* genomic epidemiology reveals the potential for zoonotic transfer. Microb Genomics. (2023) 9:1–12. doi: 10.1099/mgen.0.000974, PMID: 37000493 PMC10132062

[6] Prescott JF Mathews K Gyles CL Matsumiya L Miller C Rinkhardt N. Canine streptococcal toxic shock syndrome in Ontario: an emerging disease? Can Vet J. (1995) 36:486–7. Available at: https://pmc.ncbi.nlm.nih.gov/articles/PMC1687011/7585432 PMC1687011

[7] Matsuu A Kanda T Sugiyama A Murase T Hikasa Y. Mitral stenosis with bacterial myocarditis in a cat. J Vet Med Sci. (2007) 69:1171–4. doi: 10.1292/jvms.69.1171, PMID: 18057833

[8] Kruger EF Byrne BA Pesavento P Hurley KF Lindsay LL Sykes JE. Relationship between clinical manifestations and pulsed-field gel profiles of *Streptococcus canis* isolates from dogs and cats. Vet Microbiol. (2010) 146:167–71. doi: 10.1016/j.vetmic.2010.04.026, PMID: 20605376

[9] Eibl C Baumgartner M Urbantke V Sigmund M Lichtmannsperger K Wittek T. An outbreak of subclinical mastitis in a dairy herd caused by a novel *Streptococcus canis* sequence type (ST55). Animals. (2021) 11:550. doi: 10.3390/ani11020550, PMID: 33672442 PMC7923261

[10] Amsallem M Iung B Bouleti C Armand-Lefevre L Eme A-L Touati A. First reported human case of native mitral infective endocarditis caused by *Streptococcus canis*. Can J Cardiol. (2014) 30:1462.e1–2. doi: 10.1016/j.cjca.2014.07.013 25442453

[11] Lacave G Coutard A Troché G Augusto S Pons S Zuber B. Endocarditis caused by *Streptococcus canis*: an emerging zoonosis? Infection. (2016) 44:111–4. doi: 10.1007/s15010-015-0809-3, PMID: 26104727

[12] Wang MS Huaringa ME Feld LN Ochiai K Whelan TE Frazier NM. *Streptococcus canis* native aortic valve endocarditis linked to cat exposure: a case report and review. J Community Hosp Intern Med Perspect. (2024) 14:91–5. doi: 10.55729/2000-9666.1318, PMID: 38966509 PMC11221446

[13] Richards VP Zadoks RN Pavinski Bitar PD Lefébure T Lang P Werner B. Genome characterization and population genetic structure of the zoonotic pathogen, *Streptococcus canis*. BMC Microbiol. (2012) 12:1–16. doi: 10.1186/1471-2180-12-293, PMID: 23244770 PMC3541175

[14] Saleh E O’Neal A Torres E Vargas L Rodriguez M Chaudhary S. *Streptococcus canis* rapidly progressive and fatal neonatal Sepsis in a term infant. Pediatr Infect Dis J. (2025) 44:e161–5. doi: 10.1097/INF.0000000000004723, PMID: 39831371

[15] Imanishi I Iyori K Také A Asahina R Tsunoi M Hirano R. Antibiotic-resistant status and pathogenic clonal complex of canine *Streptococcus canis*-associated deep pyoderma. BMC Vet Res. (2022) 18:395. doi: 10.1186/s12917-022-03482-3, PMID: 36352470 PMC9644607

[16] Fulde M Rohde M Polok A Preissner KT Chhatwal GS Bergmann S. Cooperative plasminogen recruitment to the surface of *Streptococcus canis* via M protein and enolase enhances bacterial survival. MBio. (2013) 4:e00629–12. doi: 10.1128/mBio.00629-12, PMID: 23481605 PMC3604778

[17] Fulde M Rohde M Hitzmann A Preissner KT Nitsche-Schmitz DP Nerlich A. SCM, a novel M-like protein from *Streptococcus canis*, binds (mini)-plasminogen with high affinity and facilitates bacterial transmigration. Biochem J. (2011) 434:523–35. doi: 10.1042/BJ20101121, PMID: 21210764

[18] Bergmann S Eichhorn I Kohler TP Hammerschmidt S Goldmann O Rohde M. SCM, the M protein of *Streptococcus canis* binds immunoglobulin G. Front Cell Infect Microbiol. (2017) 7:80. doi: 10.3389/fcimb.2017.00080, PMID: 28401063 PMC5368172

[19] Kawara Y Goto M Maeda T Yoshida H Tsuyuki Y Takahashi T. Seven draft genome sequences of *Streptococcus canis* strains, revealing reduced penicillin-G susceptibility. Microbiol Resour Announc. (2024) 13:e0021924. doi: 10.1128/mra.00219-24, PMID: 38742884 PMC11237781

[20] Sykes JE Kittleson MD Pesavento PA Byrne BA MacDonald KA Chomel BB. Evaluation of the relationship between causative organisms and clinical characteristics of infective endocarditis in dogs: 71 cases (1992-2005). J Am Vet Med Assoc. (2006) 228:1723–34. doi: 10.2460/javma.228.11.1723, PMID: 16740074

[21] Pinho MD Matos SC Pomba C Lübke-Becker A Wieler LH Preziuso S. Multilocus sequence analysis of *Streptococcus canis* confirms the zoonotic origin of human infections and reveals genetic exchange with *Streptococcus dysgalactiae* subsp. equisimilis. J Clin Microbiol. (2013) 51:1099–109. doi: 10.1128/JCM.02912-12, PMID: 23345291 PMC3666782

[22] Taniyama D Abe Y Sakai T Kikuchi T Takahashi T. Human case of bacteremia caused by *Streptococcus canis* sequence type 9 harboring the scm gene. IDCases. (2017) 7:48–52. doi: 10.1016/j.idcr.2017.01.002, PMID: 28180088 PMC5295620

[23] Ahmad Y Gertz REJ Li Z Sakota V Broyles LN Van Beneden C. Genetic relationships deduced from emm and multilocus sequence typing of invasive *Streptococcus dysgalactiae* subsp. equisimilis and *S. canis* recovered from isolates collected in the United States. J Clin Microbiol. (2009) 47:2046–54. doi: 10.1128/JCM.00246-09, PMID: 19386831 PMC2708495

[24] Miller CS Handley KM Wrighton KC Frischkorn KR Thomas BC Banfield JF. Short-read assembly of full-length 16S amplicons reveals bacterial diversity in subsurface sediments. PLoS One. (2013) 8:e56018. doi: 10.1371/journal.pone.0056018, PMID: 23405248 PMC3566076

[25] CLSI. CLSI M100 – Performance Standards for Antimicrobial Susceptibility Testing – 34th Edition. *Clin Lab Stand Inst* (2024)

[26] CLSI. CLSI VET01S – Performance Standards for Antimicrobial Disk and Diluition Susceptibility Tests for Bacteria Isolated From Animals – 7th Edition. *Clin Lab Stand Inst* (2024)

[27] Magiorakos AP Srinivasan A Carey RB Carmeli Y Falagas ME Giske CG. Multidrug-resistant, extensively drug-resistant and pandrug-resistant bacteria: an international expert proposal for interim standard definitions for acquired resistance. Clin Microbiol Infect. (2012) 18:268–81. doi: 10.1111/j.1469-0691.2011.03570.x, PMID: 21793988

[28] Chen S Zhou Y Chen Y Gu J. Fastp: an ultra-fast all-in-one FASTQ preprocessor. Bioinformatics. (2018) 34:i884–90. doi: 10.1093/bioinformatics/bty560, PMID: 30423086 PMC6129281

[29] Prjibelski A Antipov D Meleshko D Lapidus A Korobeynikov A. Using SPAdes De novo assembler. Curr Protoc Bioinforma. (2020) 70:e102. doi: 10.1002/cpbi.102, PMID: 32559359

[30] Quast C Pruesse E Yilmaz P Gerken J Schweer T Yarza P. The SILVA ribosomal RNA gene database project: improved data processing and web-based tools. Nucleic Acids Res. (2013) 41:D590–6. doi: 10.1093/nar/gks1219, PMID: 23193283 PMC3531112

[31] Seemann T. Prokka: rapid prokaryotic genome annotation. Bioinformatics. (2014) 30:2068–9. doi: 10.1093/bioinformatics/btu153, PMID: 24642063

[32] Page AJ Cummins CA Hunt M Wong VK Reuter S Holden MTG. Roary: rapid large-scale prokaryote pan genome analysis. Bioinformatics. (2015) 31:3691–3. doi: 10.1093/bioinformatics/btv421, PMID: 26198102 PMC4817141

[33] Price MN Dehal PS Arkin AP. FastTree 2 – approximately maximum-likelihood trees for large alignments. PLoS One. (2010) 5:e9490. doi: 10.1371/journal.pone.0009490, PMID: 20224823 PMC2835736

[34] Fukushima Y Oshiteru M Yukie K Yuzo T Haruno Y Tetsuya M. Draft genome sequence of blood-origin *Streptococcus canis* strain FU149, isolated from a dog with necrotizing soft tissue infection. Microbiol Resour Announc. (2020) 9. doi: 10.1128/mra.00737-20, PMID: 32855249 PMC7453285

[35] Lefébure T Richards VP Lang P Pavinski-Bitar P Stanhope MJ. Gene repertoire evolution of *Streptococcus pyogenes* inferred from phylogenomic analysis with Streptococcus canis and *Streptococcus dysgalactiae*. PLoS One. (2012) 7:e37607. doi: 10.1371/journal.pone.0037607, PMID: 22666370 PMC3364286

[36] DeWinter LM Low DE Prescott JF. Virulence of *Streptococcus canis* from canine streptococcal toxic shock syndrome and necrotizing fasciitis. Vet Microbiol. (1999) 70:95–110. doi: 10.1016/S0378-1135(99)00128-5, PMID: 10591501

[37] Courtney HS Li Y Dale JB Hasty DL. Cloning, sequencing, and expression of a fibronectin/fibrinogen-binding protein from group a streptococci. Infect Immun. (1994) 62:3937–46. doi: 10.1128/iai.62.9.3937-3946.1994, PMID: 8063411 PMC303051

[38] Lee IPA Andam CP. Frequencies and characteristics of genome-wide recombination in *Streptococcus agalactiae*, Streptococcus pyogenes, and *Streptococcus suis*. Sci Rep. (2022) 12:1515. doi: 10.1038/s41598-022-04995-5, PMID: 35087075 PMC8795270

[39] Fuursted K Stegger M Hoffmann S Lambertsen L Andersen PS Deleuran M. Description and characterization of a penicillin-resistant *Streptococcus dysgalactiae* subsp. equisimilis clone isolated from blood in three epidemiologically linked patients. J Antimicrob Chemother. (2016) 71:3376–80. doi: 10.1093/jac/dkw320, PMID: 27585966

[40] Moyaert H Morrissey I De Jong A El Garch F Klein U Ludwig C. Antimicrobial susceptibility monitoring of bacterial pathogens isolated from urinary tract infections in dogs and cats across Europe: ComPath results. Microb Drug Resist. (2017) 23:391–403. doi: 10.1089/mdr.2016.0110, PMID: 28384093

[41] El Garch F Youala M Simjee S Moyaert H Klee R Truszkowska B. Antimicrobial susceptibility of nine udder pathogens recovered from bovine clinical mastitis milk in Europe 2015–2016: VetPath results. Vet Microbiol. (2020) 245:108644. doi: 10.1016/j.vetmic.2020.108644, PMID: 32456822

[42] Oh SI Kim JW Kim J So B Kim B Kim HY. Molecular subtyping and antimicrobial susceptibility of *Streptococcus dysgalactiae* subspecies equisimilis isolates from clinically diseased pigs. J Vet Sci. (2020) 21:e57. doi: 10.4142/jvs.2020.21.e57, PMID: 32735095 PMC7402932

[43] Rojo-Bezares B Toca L Azcona-Gutiérrez JM Ortega-Unanue N Toledano P Sáenz Y. *Streptococcus dysgalactiae* subsp. equisimilis from invasive and non-invasive infections in Spain: combining epidemiology, molecular characterization, and genetic diversity. Eur J Clin Microbiol Infect Dis. (2021) 40:1013–21. doi: 10.1007/s10096-020-04119-9, PMID: 33392783

[44] Galpérine T Cazorla C Blanchard E Boineau F Ragnaud J-M Neau D. *Streptococcus canis* infections in humans: retrospective study of 54 patients. J Infect. (2007) 55:23–6. doi: 10.1016/j.jinf.2006.12.013, PMID: 17320186

[45] Lyskova P Vydrzalova M Mazurova J. Identification and antimicrobial susceptibility of bacteria and yeasts isolated from healthy dogs and dogs with otitis externa. J Vet Med A Physiol Pathol Clin Med. (2007) 54:559–63. doi: 10.1111/j.1439-0442.2007.00996.x, PMID: 18045339

[46] Stefańska I Kwiecień E Kizerwetter-Świda M Chrobak-Chmiel D Rzewuska M. Tetracycline, macrolide and lincosamide resistance in *Streptococcus canis* strains from companion animals and its genetic determinants. Antibiotics. (2022) 11. doi: 10.3390/antibiotics11081034, PMID: 36009903 PMC9405182

[47] Fukushima Y Tsuyuki Y Goto M Yoshida H Takahashi T. Novel quinolone nonsusceptible *Streptococcus canis* strains with point mutations in quinolone resistance-determining regions and their related factors. Jpn J Infect Dis. (2020) 73:242–9. doi: 10.7883/yoken.JJID.2019.392, PMID: 32009056

[48] Pinho MD Foster G Pomba C MacHado MP Baily JL Kuiken T. *Streptococcus canis* are a single population infecting multiple animal hosts despite the diversity of the universally present M-like protein SCM. Front Microbiol. (2019) 10:1–10. doi: 10.3389/fmicb.2019.00631 30984150 PMC6450190

[49] Fukushima Y Takahashi T Goto M Yoshida H Tsuyuki Y. Novel diverse sequences of the *Streptococcus canis* M-like protein (SCM) gene and their prevalence in diseased companion animals: association of their alleles with sequence types. J Infect Chemother. (2020) 26:908–15. doi: 10.1016/j.jiac.2020.04.004, PMID: 32354600

[50] Yoshida H Goto M Fukushima Y Maeda T Tsuyuki Y Takahashi T. Intracellular invasion ability and associated microbiological characteristics of *Streptococcus canis* in isolates from Japan. Jpn J Infect Dis. (2021) 74:129–36. doi: 10.7883/yoken.JJID.2020.382, PMID: 32863352

